# *Listeria monocytogenes* Response to Sublethal Chlorine Induced Oxidative Stress on Homologous and Heterologous Stress Adaptation

**DOI:** 10.3389/fmicb.2018.02050

**Published:** 2018-08-31

**Authors:** Mohit Bansal, Ramakrishna Nannapaneni, Chander S. Sharma, Aaron Kiess

**Affiliations:** ^1^Department of Poultry Science, Mississippi State University, Starkville, MS, United States; ^2^Department of Food Science, Nutrition and Health Promotion, Mississippi State University, Starkville, MS, United States

**Keywords:** *Listeria monocytogenes*, sublethal oxidative stress, chlorine, sodium hypochlorite, stress adaptation

## Abstract

The objective of this study was to determine the effect of chlorine induced sublethal oxidative stress against homologous and heterologous stress adaptations in five *Listeria monocytogenes* (*Lm*) strains. *Lm* cells were exposed to gradually increasing sublethal concentrations of total chlorine/day: 250 ppm (day 1), 270 ppm (day 2), 290 ppm (day 3), 310 ppm (day 4), 330 ppm (day 5), 350 ppm (day 6), and 375 ppm (day 7) in tryptic soy broth (TSB). Changes in minimum inhibitory concentration (MIC) and minimum bactericidal concentration (MBC) of *Lm* cells exposed to chlorine and control (non-adapted cells) were determined by the macro-dilution method. Chlorine-adapted *Lm* cells were also evaluated for changes in antibiotic resistance using the Kirby–Bauer disk diffusion and MIC double dilution assay as per the Clinical and Laboratory Standards Institute (CLSI, 2016) guidelines. In four *Lm* strains (Scott A, V7, FSL-N1-227 and FSL-F6-154) after adapted to sublethal chlorine, the MIC (600 ppm) and MBC (700 ppm) values of chlorine were slightly higher as compared to control (500 ppm MIC, and 600 ppm MBC). The Kirby–Bauer and MIC double dilution assays showed some significant changes in antibiotic susceptibility patterns for antibiotics such as streptomycin, gentamicin and ceftriaxone (*p* < 0.05). However, the changes in zones of inhibition and MIC values to all antibiotics tested for the chlorine-adapted and non-adapted (control) *Lm* cells were still within the susceptible range. Transmission electron microscopy studies showed that changes in cell wall and membrane integrity resulting, from the elongation of cells, may contribute to the possible routes of its increase in tolerance to chlorine and selective antibiotics. These findings indicate that the continuous exposure of *Lm* cells to chlorine may lead to significant changes in homologs and heterologous stress adaptation.

## Introduction

*Listeria monocytogenes* is a ubiquitous Gram-positive foodborne bacterial pathogen. *Listeriosis*, caused by *Listeria monocytogenes*, is a fatal foodborne disease with a high hospitalization rate (>90%), and commonly causes an infection in susceptible populations which include immunocompromised individuals and pregnant women (Scallan et al., 2011). Though *listeriosis* has a low incidence rate (<3%) in the healthy population, it is the leading cause of foodborne related deaths and causes 250 deaths annually in the United States (Mead et al., 1999; CDC, 2012). This foodborne pathogen causes severe nervous symptoms such as meningitis, meningoencephalitis (invasive listeriosis) and febrile gastroenteritis (non-invasive listeriosis) in the susceptible population (Allerberger and Wagner, 2010). Often, non-invasive listeriosis the cases are commonly treated with antibiotics such as ampicillin, amoxicillin, gentamicin, or cephalosporin. However, a few strains of *L. monocytogenes* have been found to have intrinsic resistance against third generation cephalosporin drugs (Kose and Yakupogullari, 2015).

In the food-processing environments, *L. monocytogenes* is commonly exposed to oxidative stress produced by sanitizers and disinfectants or antimicrobial rinses like chlorine (Gao and Liu, 2014; Gray et al., 2014). In the food-production environments, indiscriminate and over usage of antibiotics has raised concerns over the emergence of antibiotic resistance (Fair and Tor, 2014). Since the 1960s, there has been a gradual increase in the number of antibiotic resistant cases from all over the world with treatment costs exceeding $20 billion dollars annually (Fair and Tor, 2014). Other published findings show that continuous exposure of bacteria to disinfectants does not inhibit the complete metabolic and/or genetic activity of bacteria and may induce the phenotypic resistance mechanism which is expressed in the form of changes in cell wall and membrane structures (Wiuff et al., 2005; McMahon et al., 2007) or altered activity of specific or non-specific efflux pumps (Levin and Rozen, 2006).

Biocides are commonly used for routine cleaning, sanitation and disinfection in the food industry and hospitals (Holah et al., 2002; Seier-Petersen, 2013). Biocide’s lethal concentration might reduce to sublethal concentration in food processing plant especially in places where water and organic matter are abundant (Carpentier and Cerf, 2011). Continuous exposure to sublethal concentrations of these biocides/antimicrobials while cleaning and sanitizing process may induce stress adaptation or response in food borne pathogens (Capozzi et al., 2009). Previous studies reported that the persistence of certain strain of *L. monocytogenes* in food processing environment is not a random phenomenon but directly related with different situation which results in sublethal conditions such as inefficient cleaning before disinfection, disinfection of wet surface or dosage failure (Martínez-Suárez et al., 2016). In response to sublethal biocide stress, bacteria can co-select genes responsible for encoding tolerance to both same or different biocides and antibiotics (Henshaw and O’Carroll, 2009). In a previous study, Romero et al. (2017) found significant correlation between occurrence of antibiotic and biocide resistance in bacterial isolates from seafood.

Phenotypic adaptations, a form of temporary or sustained antibiotic tolerance, in response to sublethal stresses include changes in the permeability of the cell membrane, and increased expression of specific or non-specific efflux pumps (Forbes et al., 2014). McMahon et al. (2007) found that exposure of low pH or high NaCl concentrations can trigger the development of antibiotic resitance in subpopulations of pathogens. Apart from other factors, bile acids also have the capacity to induce oxidative stress adaptation in *L. monocytogenes*. Increased expression of bile salt hydrolase by the bacterium in response to bile salt exposure can aid in *L. monocytogenes* survival (Begley et al., 2005). Later, Gadea et al. (2017) observed repeated exposure of bacteria to QAC biocide can co-select for antibiotic tolerance which could be temporary or sustained.

There is a lack of knowledge on the influence of continuously increasing doses of sublethal chlorine for short term exposure on *L. monocytogenes* homologous adaptation (tolerance to same biocide) and heterologous adaptation (cross-resistance to other biocides and antibiotics). Therefore, three objectives were addressed in this study: (i) to determine homologous stress adaptation to chlorine in *L. monocytogenes* strains by measuring the changes in minimum inhibitory concentration (MIC) and minimum bactericidal concentration (MBC); (ii) to determine the cross-resistance to commonly used antibiotics by measuring changes in zones of inhibition and MIC in *L. monocytogenes* cells after exposure to sublethal chlorine; (iii) to determine ultrastructural changes in *L. monocytogenes* after exposure to sublethal chlorine by transmission electron microscopy.

## Materials and Methods

### Bacterial Strains and Culture Preparation

Five *L. monocytogenes* strains used in this study are listed in **Table 1**. All bacterial strains were stored as stock cultures in tryptic soy broth (TSB) supplemented with 25% glycerol at -80°C. This bacteriological media, Difco (Becton Dickinson, Sparks, MD, United States) was used for all experiments in the present study. Prior to each experiment, individual bacterial strains were cultured in 10 ml of TSB at 37°C for two consecutives 24 h cycles to remove any cold stress in working cultures. Following overnight incubation, the culture was centrifuged at 5000 × *g* for 10 min at 4°C and the collected pellet resuspended in 10 ml of TSB (pH 7.2). Serial 10-fold dilutions were plated on duplicate tryptic soy agar (TSA) and modified oxford agar plates for enumeration and confirmation, respectively. The plates were analyzed for CFU after 24 h incubation at 37°C.

**Table 1:** List of *Listeria monocytogenes* strains and their sources used in this study.

No.	*L. monocytogenes* strains	Lineage	Serovar	First reported outbreak
(1)	*L. monocytogenes* FSL F6-154	II	1/2a	Food, epidemic (sliced turkey) (2000)
(2)	*L. monocytogenes* FSL N1-227	I	1/2a	Food, epidemic (US 1998–1999)
(3)	*L. monocytogenes* ATCC 19116	III	4c	Poultry, UW
(4)	*L. monocytogenes* Scott A	I	4b	Human epidemic (Mass, 1983)
(5)	*L. monocytogenes* V 7	II	1/2a	Raw milk, FDA

### Sodium Hypochlorite

A 5% stock solution of Sodium hypochlorite (Acros Organics, New Jersey, United States) was used as the source of chlorine. The stock solution was used to prepare the working concentrations of chlorine in TSB. Prior to each experiment, total and free available chlorine was measured using an HACH (chlorine test kit) Pocket Colorimeter (HACH Company, Loveland, CO, United States).

### Minimum Inhibitory Concentration (MIC) and Minimum Bactericidal Concentration (MBC) Assay

The MIC was determined by the macro-dilution method with minor modifications of the Clinical and Laboratory Standards Institute (CLSI, 2016). The overnight culture of *L. monocytogenes* at 10^9^ CFU/ml was diluted in TSB to obtain a final concentration of 10^6^ CFU/ml for the MIC assay. The total available chlorine concentration range of 200–800 ppm, with 100 ppm increments, was prepared in 10 ml volumes of TSB. An aliquot (100 μL) of *L. monocytogenes* suspension (10^6^ CFU/ml) was added to each tube containing working chlorine dilutions to observe bacterial growth by turbidity changes after incubation at 37°C for 24 h. Tubes with *L. monocytogenes* inoculum without chlorine and tubes with TSB alone were used as a positive and negative control, respectively. MIC was determined to be the lowest concentration of available chlorine which inhibited the visible growth of *L. monocytogenes* after 24 h of incubation.

Aliquots of 100 μL from tubes without visible growth of *L. monocytogenes* were spread plated onto duplicate TSA plates to determine the minimum bactericidal concentration (concentration of available chlorine that kills all *L. monocytogenes* cells after 24 h incubation at 37°C).

### Chlorine Induced Oxidative Stress Adaptation in *Listeria monocytogenes*

The objective of this study to evaluate the sublethal chlorine induced oxidative stress on homologous and heterologous tolerance in *L. monocytogenes* under laboratory condition that simulated to food processing plant. The approach was abusive disinfection, or cleaning procedure or abundance of water and organic matter in food processing environment may responsible for sublethal chlorine exposure to *L. monocytogenes*. A modified sublethal chlorine adaptation protocol for a short period of 7 days was used in the present study. An aliquot (100 μL) of a 24 h previously prepared bacterial culture, was added to 10 ml of TSB with the initial 250 ppm chlorine concentration (day 1). Subsequently 100 μl (∼10^7^ CFU/ml) of a previous day incubated culture (250 ppm) after 24 h was transferred into fresh TSB with the gradually increasing chlorine concentrations of 20 ppm/day, i.e., 270 ppm (day 2), 290 ppm (day 3), 310 ppm (day 4), 330 ppm (day 5), and 350 ppm (day 6). Finally, on day 7, the final chlorine concentration of 375 ppm in TSB was obtained by increasing 25 ppm in previous day culture. Therefore, *L. monocytogenes* adapted cells from three different sublethal oxidative stress levels were obtained: (1) at 250 ppm (1/2 MIC) for 1 day, (2) by gradual exposure from 250 ppm (1/2 MIC) to 330 ppm (2/3 MIC) over 5 days; and (3) by gradual exposure from 250 ppm (1/2 MIC) to 375 ppm (3/4 MIC) over 7 days. Control (non-adapted) cells were also passaged and harvested along with the adapted cells in similar fashion but in the absence of chlorine.

### Viable Cell Counts

Sublethal chlorine adapted (250 ppm, 250–330 ppm, or 250–375 ppm) and control (non-adapted) were tested for turbidity at OD_600_ and CFU counts/ml after their overnight incubation periods. OD_600_ of both adapted and non-adapted was evaluated with spectrophotometer (Model ELx800, BioTek Instruments, Winooski, VT, United States). CFU were enumerated from serial dilution of 100 μl of overnight grown non-adapted and chlorine adapted treatments in 0.1% buffered peptone water, and aliquot of 100 μl from each dilution was plated on duplicated TSA plate and modified oxford agar plates for incubation at 37°C for 48 h.

### Determination of Chlorine Tolerance Development After Chlorine Adaptation

The chlorine adaptive tolerance was measured by determining the MIC and MBC for the control and chlorine adapted cells using broth macro dilution method (CLSI, 2016). Initial inoculum size of 10^6^ CFU/ml of control and three sublethal oxidative stress levels (250 ppm, 250–330 ppm, or 250–375 ppm) cells was inoculated in 10 mL TSB with chlorine at concentrations (500, 600, 700, and 800 ppm) that were equivalent or above the MIC to measure chlorine adaptation in terms of MIC changes and incubated at 37°C for 24 h. Tubes with no visible turbidity after incubation was considered as MIC. To assess changes in MBC, an aliquot of 100 μl from 24 h previously incubated chlorine containing TSB tubes was spread plated on duplicated TSA plate and incubated for 24 h at 37°C. Complete inhibition of *L. monocytogenes* growth on TSA agar plate from lowest chlorine concentration containing TSB tube was considered as MBC.

### Determination of Antibiotic Susceptibility

#### Antibiotic MIC Determination by Disk-Diffusion Assay

The Kirby–Bauer disk-diffusion method was used to compare the zones of inhibition of *L. monocytogenes* oxidative stress adapted cells to their controls. Cells from controls and sublethal oxidative stress levels (after exposure to 250 ppm for 1 day, 250–330 ppm over 5 days, and 250–375 ppm over 7 days) were obtained and pelleted after centrifugation at 5000 × *g* for 10 min at 4°C. Cells were resuspended in 10 ml of fresh TSB. Colonies were isolated on fresh TSA plates after incubation at 37°C for 24 h. McFarland standards were prepared in 0.1% peptone water from colonies obtained from both the control and sublethal oxidative stress adapted cells. Aliquots were swabbed on Mueller-Hinton agar and antibiotic disks were placed on the plate. The plates were incubated at 37°C for 24 h. The zones of inhibition (mm) for different antibiotics were obtained for *L. monocytogenes* cells exposed to three chlorine oxidative stress levels of 250 ppm for 1 day, 250–330 ppm over 5 days, or 250–375 ppm over 7 days and control (non-adapted) cells.

The antibiotic disks used were: amoxicillin/clavulanic acid (AMC, 30 μg), gentamicin (GEN, 10 μg), Sulfamethoxazole/trimethoprim (SXT, 25 μg), streptomycin (STR, 10 μg), nalidixic acid (NA, 30 μg), ciprofloxacin (CIP, 5 μg), ceftriaxone (CTX, 30 μg), ampicillin (AMP, 10 μg), vancomycin (VAN, 30 μg), and rifampicin (RIF, 5 μg). The zones of inhibition were measured according to the guidelines CLSI (2016).

#### Antibiotic MIC Determination by Micro-Dilution Assay

The stock solution of antibiotics was prepared as the manufactures recommended (**Table 2**). Working concentrations were obtained in Mueller-Hinton broth (MHB) from stock solutions. The MIC was determined by serial double fold dilutions for each antibiotic obtained in MHB using a 96-well round bottom sterile polystyrene microtiter plate (12 columns by 8 rows). Eight serial double dilutions were acquired in 8 columns and were duplicated in 4 rows. The MIC breakpoints recommended by the CLSI (2016) were used to observe the results as the lowest concentration of antibiotics which inhibited the visible growth as susceptible, intermediate or resistant. The susceptible range of each antibiotic is listed in **Table 2**. One well of MHB with *L. monocytogenes* inoculum and antibiotics was used as a positive control and one well with only MHB was used as the negative control. The lowest concentration of antibiotics that inhibited the button formation and yielded no turbidity was considered the MIC. The MIC values from all three sublethal chlorine oxidative stress levels (250 ppm for 1 day, 250–330 ppm over 5 days, or 250–375 ppm over 7 days) were obtained and compared to the control cells value.

**Table 2:** List of antibiotics used in this study.

No.	Antibiotic	Susceptibility range (μg/ml)	Solvent	Diluent
(1)	Ampicillin	0.0078–1	Water	MHB
(2)	Amoxicillin	0.0075–0.48	Water	MHB
(3)	Ceftriaxone	2–128	Water	MHB
(4)	Vancomycin	0.5–32	Water	MHB
(5)	Tetracycline	0.03–2	Ethanol (70%)	MHB
(6)	Gentamicin	0.062–4	Water	MHB
(7)	Streptomycin	4–256	Water	MHB
(8)	Ciprofloxacin	0.0075–0.48	Water	MHB

### Determination of Ultrastructure Changes in the Chlorine Adapted Cells by Transmission Electron Microscopy

Transmission electron microscopy (TEM) was used to investigate the ultrastructural changes in *L. monocytogenes* cells after sublethal chlorine exposure. As explained earlier, *L. monocytogenes* cells were exposed to gradually increasing concentrations of chlorine from 250 ppm (day 1) to the final concentration of 375 ppm (day 7) in TSB. *L. monocytogenes* strains, Scott A, V7 and FSL-N1-227 chlorine adapted cells were harvested after exposure to a final 375 ppm by centrifugation at 5,000 × *g* for 10 min at 4°C to remove residual chlorine and the resulting concentrated cell pellets were prepared for TEM by previously described methods (Capita et al., 2014). Pellets were fixed using 1/2 strength Karnovsky fixative in 0.1 M sodium cacodylate buffer (pH 7.2) overnight at 4°C. Fixed cells were washed in buffer, post fixed in 2% buffered osmium tetroxide, dehydrated through a graded ethanol series, and embedded in Spurr’s resin. Ultra-thin sections were cut using a Riecher Jung Ultracut microtome, collected on copper grids and stained with uranyl acetate and lead citrate and viewed on a JEOL JSM-1230 transmission electron microscope (Jeol USA, Peabody, MA, United States) at 80 kv. For the TEM analysis, four different random areas were selected to take images for each sample. Cells were classified into normal (short rods) and stress adapted (elongated rods) based on the standard length on the similar magnification scale.

### Statistical Analysis

The homologous stress adaptation assays (MIC and MBC determination to chlorine) were repeated three times. While evaluating for heterologous stress adaptation, antibiotic disk diffusion assays were repeated three times and antibiotic MIC dilution assays were repeated two times. All experimental data were analyzed using a 2 × 3 factorial arrangement (chlorine adapted and non-adapted treatments versus three stress periods) in a randomized complete block design with replication considered as block using SAS v. 9.4 (SAS Institute, Cary, NC, United States; Steel and Torrie, 1980). The means were separated using Fisher Protected Least Significance difference. The treatments and controls were determined to be significant when *P* ≤ 0.05.

## Results

### Viable Cell Count

Viable cell counts, and growth turbidity (OD_600_) were measured to investigate effect of sublethal concentrations of chlorine exposure to *L. monocytogenes* strains on growth phase. Results showed that sublethal concentrations of chlorine did not significantly change in growth rate of *L. monocytogenes* strains at 37°C. Both non-adapted control cells and chlorine adapted cells of *L. monocytogenes* reached the stationary phase during their overnight incubation periods prior to testing for homologous and heterologous adaptation.

### Homologous Stress Adaptation (Changes in Chlorine Tolerance)

Homologous stress adaptation of *L. monocytogenes* to chlorine was determined by measuring the changes in MIC and MBC for sodium hypochlorite. The MIC of sodium hypochlorite for control *L. monocytogenes* was 500 ppm for all five strains (Scott A, V7, N1-2227, F6-154, and ATCC 19116) studied. After exposure to the sublethal oxidative stress induced by chlorine, the average MIC of sodium hypochlorite was slightly increased to 600 ppm for cells at all three sublethal stress levels, i.e., 250, 330, and 375 ppm in four strains (Scott A, V7, N1-227, and F6-154). However, there was no change in MIC to sodium hypochlorite for the reference strain ATCC 19916 under these experimental conditions.

The MBC of sodium hypochlorite for *L. monocytogenes* showed similar trends of adaptive tolerance to sodium hypochlorite, where average MBC was increased to 700 ppm for strains Scott A, V7, N1-227, F6-154 from the initial 600 ppm for cells at all sublethal stress levels (250, 330, and 375 ppm). However, no change in MBC was observed for the reference strain ATCC 19116 (**Table 3**).

**Table 3:** Minimum inhibitory concentrations (MICs) and minimum bactericidal concentrations (MBCs) of chlorine (ppm) for five *L. monocytogenes* strains adapted to sublethal concentrations of chlorine versus control.

Chlorine tolerance		*L. monocytogenes* strains				
		Scott A	V7	FSL-N1-227	FSL-F6-154	ATCC 19116
MIC (ppm)	Control	500 ± 0	500 ± 0	500 ± 0	500 ± 0	500 ± 0
	Adapted	600 ± 0	600 ± 0	600 ± 0	600 ± 0	500 ± 0
MBC (ppm)	Control	600 ± 0	600 ± 0	600 ± 0	600 ± 0	600 ± 0
	Adapted	700 ± 0	700 ± 0	700 ± 0	700 ± 0	600 ± 0

*All assays repeated three times.*

### Heterologous Stress Adaptation (Changes in Antibiotic Susceptibility)

Antibiotic susceptibility of five strains of *L. monocytogenes* was measured by the Kirby–Bauer disk diffusion assay and MIC dilution assay as per CLSI (2016) guidelines. The antibiotic susceptibility patterns of the oxidative stress adapted and control cells from both the disk diffusion assay and MIC dilution method are shown in **Tables 4, 5**. *L. monocytogenes* strains were screened for susceptibility to different classes of antibiotics before and after gradual exposure to the increasing concentrations of sodium hypochlorite. All five strains tested were found to have intrinsic resistance to nalidixic acid while 2 of the five strains tested (Scott A and N1-227) have intrinsic resistance to ceftriaxone (a third-generation cephalosporin). The average zone of inhibition was slightly decreased by 0.5–2.2 mm compared to controls for all 11 antibiotics tested. Interestingly, the disk diffusion assay showed that all five-strains studied in the present study develop significant tolerance for streptomycin (*p* < 0.05). Significant antibiotic tolerance development was also observed for antibiotics such as ampicillin, amoxicillin, sulfamethoxazole and trimethoprim, ciprofloxacin, rifampicin, and ceftriaxone after sublethal chlorine exposure to *L. monocytogenes* by disk diffusion assay. Results showed that the effect of sublethal chlorine exposure on *L. monocytogenes* has significantly decreased its zone of inhibition for few of antibiotics, however, it did not influence antibiotic susceptibility zone.

**Table 4:** Average zones of inhibition (mm) by antibiotic disk-diffusion tests for five *L. monocytogenes* strains after chlorine induced oxidative stress adaptation (A) over three different stress levels (1/2 MIC for 1 day, 1/2–2/3 MIC over 5 days and 1/2–3/4 MIC over 7 days) versus control (C) cells.

Antibiotics^a^	*L. monocytogenes* strains									
	Scott A		V7		FSL-N1- 227		FSL-F6-154		ATCC-19116	
	C	A	C	A	C	A	C	A	C	A
AMP	**32 ± 0.3**	**31 ± 0.3^∗^**	**34 ± 0.3**	**31 ± 0.3^∗^**	30 ± 0.02	29 ± 0.02	**32 ± 0.2**	**30 ± 0.2^∗^**	32 ± 0.3	32 ± 0.3
STR	**20 ± 0.3**	**18 ± 0.3^∗^**	**19 ± 0.4**	**18 ± 0.4^∗^**	**19 ± 0.2**	**17 ± 0.2^∗^**	**20 ± 0.3**	**18 ± 0.3^∗^**	**20 ± 0.2**	**18 ± 0.2^∗^**
GEN	22 ± 0.3	22 ± 0.3	22 ± 0.2	22 ± 0.2	22 ± 0.3	21 ± 0.3	22 ± 0.4	21 ± 0.4	23 ± 0.4	23 ± 0.4
AMC	34 ± 0.1	34 ± 0.1	34 ± 0.5	33 ± 0.5	**34 ± 0.3**	**33 ± 0.3^∗^**	**34 ± 0.2**	**30 ± 0.2^∗^**	**34 ± 0.2**	**32 ± 0.2^∗^**
NA	0^b^	0^b^	0^b^	0^b^	0^b^	0^b^	0^b^	0^b^	0^b^	0^b^
SXT	**36 ± 0.2**	**35 ± 0.2^∗^**	34 ± 0.3	34 ± 0.3	35 ± 0.4	33 ± 0.4	**35 ± 0.3**	**32 ± 0.3^∗^**	**34 ± 0.3**	**31 ± 0.3^∗^**
VAN	**22 ± 0.3**	**21 ± 0.3^∗^**	22 ± 0.3	22 ± 0.3	22 ± 0.3	21 ± 0.3	22 ± 1.0	21 ± 1.0	**23 ± 0.2**	**22 ± 0.2^∗^**
CIP	21 ± 1.0	20 ± 1.0	22 ± 0.2	23 ± 0.2	22 ± 0.3	22 ± 0.3	22 ± 0.4	21 ± 0.4	**22 ± 1.0**	**21±1.0^∗^**
RIF	**24 ± 0.3**	**25 ± 0.3^∗^**	25 ± 1.0	24 ± 1.0	**25 ± 0.2**	**24 ± 0.2^∗^**	24 ± 0.3	25 ± 0.29	25 ± 0.3	24 ± 0.3
CTX	0^b^	0^b^	14 ± 0.3	13 ± 0.3	0^b^	0^b^	14 ± 0.3	13 ± 0.3	**14 ± 0.2**	**12 ± 0.2^∗^**

*All assays were repeated three times.*

*^*a*^AMP, ampicillin; STR, streptomycin; GEN, gentamicin; AMC, amoxicillin/clavulanic acid; NA, nalidixic acid; SXT, sulfamethoxazole and trimethoprim; VAN, vancomycin; CIP; ciprofloxacin; RIF’, rifampicin; CTX, ceftriaxone. ^*b*^Possess intrinsic resistance to NA and CTX. Zones of inhibition****±****standard error showing significant differences between control and adapted cells are highlighted in bold. ^∗^Indicate p < 0.05*.

Similarly, development of antibiotic tolerance was measured after sublethal chlorine exposure to *L. monocytogenes* with double dilution MIC assay. The assay showed that significant antibiotic tolerance was developed for ampicillin (one strain), tetracycline (two strains), streptomycin (one strain), and ceftriaxone (three strains). A major change in MIC was observed for the reference strain ATCC 19116 which exhibited a fourfold increase in MIC to ceftriaxone after sublethal chlorine adaptation (**Table 5**). A minor but significant change in antibiotic susceptibility range for ceftriaxone was also observed in two intrinsic resistant strains, Scott A and N1-227, in chlorine oxidative stress adapted cells of *L. monocytogenes.* The decrease in zone of inhibition and increase in MIC for antibiotics after sublethal chlorine exposure could be related with phenotypic antibiotic tolerance development in *L. monocytogenes*.

**Table 5:** Average minimum inhibitory concentrations (MICs) of antibiotics for five *L. monocytogenes* strains after chlorine induced oxidative stress adaptation (A) over three different stress levels (1/2 MIC for 1 day, 1/2–2/3 MIC over 5 days and 1/2–3/4 MIC over 7 days) versus control (C).

Antibiotics^a^	*L. monocytogenes* strains									
	Scott A		V7		FSL-N1- 227		FSL-F6-154		ATCC-19116	
	
	C	A	C	A	C	A	C	A	C	A
AMP	0.3 ± 0.04	0.3 ± 0.04	**0.08 ± 0.01**	**0.2 ± 0.01^∗^**	0.5 ± 0.09	0.6 ± 0.09	0.25 ± 0.0	0.25 ± 0.0	0.3 ± 0.06	0.4 ± 0.06
TET	**0.3 ± 0.02**	**0.5 ± 0.02^∗^**	**0.3 ± 0.04**	**0.5 ± 0.04^∗^**	1.0 ± 0.2	0.9 ± 0.2	1.12 ± 0.0	1.12 ± 0.0	0.5 ± 0.3	0.6 ± 0.26
STR	3.5 ± 0.0	3.5 ± 0.0	**7 ± 0.0**	**14 ± 0.0^∗^**	15 ± 1.2	15 ± 1.2	37 ± 1.0	40 ± 1.0	10 ± 1.5	15 ± 1.53
GEN	0.3 ± 0.07	0.3 ± 0.07	0.5 ± 0.0	1 ± 0.0	1.5 ± 0.01	2 ± 0.01	1.7 ± 0.2	1.8 ± 0.2	1.4 ± 0.35	1.6 ± 0.35
AMX	0.1 ± 0.02	0.14 ± 0.02	0.12 ± 0.1	0.15 ± 0.01	0.4 ± 0.0	0.4 ± 0.0	0.2 ± 0.03	0.3 ± 0.03	0.2 ± 0.0	0.2 ± 0.0
VAN	0.5 ± 0.0	0.5 ± 0.0	0.5 ± 0.0	0.5 ± 0.0	0.5 ± 0.0	0.5 ± 0.0	0.5 ± 0.0	0.5 ± 0.0	0.5 ± 0.0	0.5 ± 0.0
CIP	0.3 ± 0.0	0.3 ± 0.0	0.3 ± 0.0	0.3 ± 0.0	0.3 ± 0.0	0.5 ± 0.0^∗^	0.4 ± 0.0	0.5 ± 0.0	0.5 ± 0.0	0.5 ± 0.0
CTX	**17 ± 3.0**	**29 ± 3.0^∗^**	7.3 ± 0.59	7 ± 0.59	**19 ± 1.8**	**27 ± 1.8^∗^**	6.6 ± 0.0	8 ± 0.0	8 ± 0.0	32 ± 0.0

*All assays repeated two times.*

*^*a*^AMP, ampicillin; TET, tetracycline; STR, streptomycin; GEN, gentamicin; AMX, amoxicillin; VAN, Vancomycin; CIP; ciprofloxacin; CFT, Ceftriaxone. MIC****±****standard error showing significant differences between control and adapted cells are highlighted in bold. ^∗^Indicate p < 0.05.*

### Changes in Ultrastructure of *L. monocytogenes* in the Presence of Sodium Hypochlorite

**FIGURE 1:**
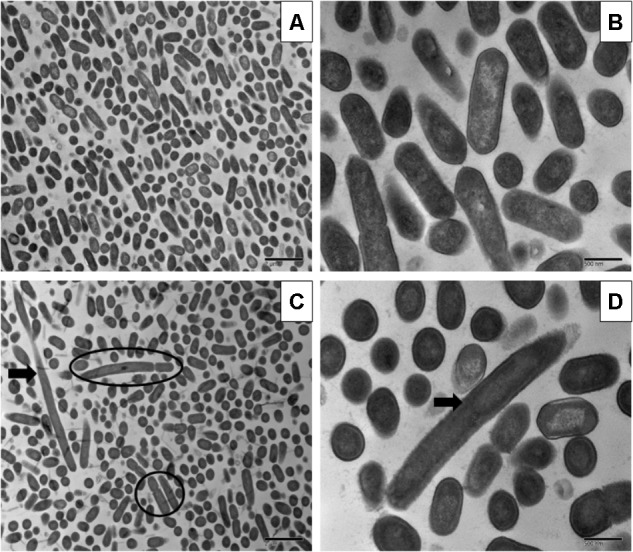
Transmission electron micrographs of *Listeria monocytogenes* Scott A non-adapted **(A,B)** and oxidative stress adapted cells **(C,D)** after gradually exposing to 375 ppm (3/4 MIC) from 250 ppm (1/2 MIC) of chlorine over 7 days. Micrographs represent planktonic cells: **(A,B)** control cells at different magnifications; **(C,D)** elongated cells and bud formation, indicated by arrow and multi-chromosome formation indicated by red circle in chlorine adapted cells at different magnifications.

**FIGURE 2:**
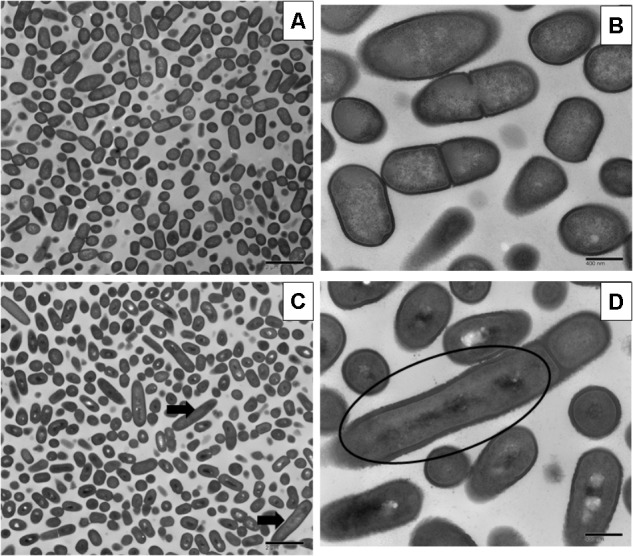
Transmission electron micrographs of *L. monocytogenes* V7 non-adapted **(A,C)** and oxidative stress adapted cells **(B,D)** gradually exposing to 375 ppm (3/4 MIC) from 250 ppm (1/2 MIC) of chlorine over 7 days. Micrographs represent planktonic cells: **(A,B)** control cells at different magnifications; **(C,D)** elongated cells and bud formation indicated by arrow, and multi-chromosome formation and inhibition of cell division indicated by red circle in chlorine adapted cells at different magnifications.

Sublethal chlorine induced oxidative stress caused significant and consistent morphological changes in *L. monocytogenes* strains Scott A, V7 and N1-227 which included: (1) changes in cell length, (2) changes in cell wall thickness, (3) changes in cell membrane shape; and (4) cytoplasmic changes. Sublethal chlorine adapted *L. monocytogenes* exhibited a consistent elongation of cells, presence of multiple chromosomes and membrane bleb formations which may indicate transient inhibition of cell division (**Figures 1, 2** and **Supplementary Figure S1**).

Along with the elongated cells, outer membrane bleb formation and wavy cell envelop structures were frequently observed in chlorine stress adapted cells of *L. monocytogenes* which may be associated with the activation of a bacterial phenotypic response to sublethal oxidative stress.

## Discussion

The objective of this study was to evaluate the influence of sublethal concentrations of sodium hypochlorite on homologous and heterologous stress adaptation in *L. monocytogenes* in laboratory condition simulating food processing environment. Various sanitizers and disinfectants are routinely used at >1,000 times concentrations than that of their MIC for killing foodborne pathogens in the food-processing plants. The high concentrations of sanitizers and disinfectants interacts with multiple mechanisms in bacterial cells, such as degeneration of proteins, and lipids, as well as DNA degradation (Gray et al., 2014). In food processing environments, bacterial cells are frequently exposed to lower or sublethal concentrations of biocides because of dilution of biocide by water abundance and presence of organic matter. Similar conditions of sublethal exposure are also occurred in wastewater plants, hospitals, or in processing plant effluents. Recent findings show that the sublethal concentrations of biocides interact with a single central bacterial response and such gradual exposure of sublethal concentrations can co-select resistance to biocides as well as to antibiotics (Capita et al., 2014; Gray et al., 2014; Wales and Davies, 2015; Webber et al., 2015). Therefore, it is important to understand the role of sublethal concentrations at which biocides can select or co-select for antibiotic resistance in whole or subpopulations of bacterial cells.

*Listeria monocytogenes* strains Scott A, V7, N1-227, and F6-154 exhibited homologous stress adaptation in TSB with a 100-ppm increase in chlorine tolerance as evidenced by increase in MIC and MBC. In other studies, Kastbjerg and Gram (2012) exposed *L. monocytogenes* EGD to the disinfectants hypochlorite and Incimaxx DES (peracetic acid and hydrogen peroxide) for several hundred generations (300–420 bacterial generations) and found no changes in the MIC, whereas exposure to Triquart SUPER (quaternary ammonium compounds) caused a two–fourfold increase in MIC. However, in the present study MIC was determined at a close range of 500, 600, 700, and 800 ppm chlorine concentrations after 1, 5, or 7 serial transfers which showed that development of homologous stress adaptation after serial transfer in presence of sublethal chlorine concentrations. Chlorine reacts with organic compounds in media (TSB) and forms a reactive chlorine species (RCS) such as chloramines. The RCS then reacts with sulfur containing compounds such as amino acids (cysteine, methionine or glutathione), and primary or secondary amines, nucleotides and lipids. While the higher concentration of RCS damages the bacterial DNA, bacteria can generate adaptations when exposed to a lower or sublethal concentration. Bacteria quickly respond to RCS by the high expression of enzymes, such as catalases, peroxidases, and methionine sulfoxide reductase. In response to damaged DNA caused by RCS, bacteria upregulate homologous recombination, repair and mutagenic polymerases (Gray et al., 2014).

Antibiotic susceptibility is measured by disk diffusion, *E*-test and broth microdilution assay. The broth microdilution method is the primarily standard reference method for quantifying antibiotic susceptibility/resistance responses in clinical and research labs (CLSI, 2016). This method is a technically simple, fast, and low-cost method compared to E-test (Luber et al., 2003). In the present study, Kirby–Bauer disk diffusion and broth microdilution assay was used for analysis of antibiotic susceptibility. For clinical purpose, drug dosage is a function of MIC, half-life and distribution volume of antibiotic. MIC is an important factor in calculation of antibiotic dose and increase in MIC will decrease pharmacodynamics index and result in unlikely clinical response (MacGowan, 2008). In present study, heterologous stress adaptation results showed that *L. monocytogenes* developed minor but statistically significant increase in MICs for commonly used antibiotics which may impact on increase of antibiotic dosage and also causes antibiotic resistance.

The antibiotic resistance against third generation cephalosporins in *L. monocytogenes* was reported from different countries. Ceftriaxone resistance in human listeriosis cases were reported from Vietnam (Chau et al., 2010), Bangladesh (Ahmed et al., 2015), and Turkey (Kose and Yakupogullari, 2015). Strains of a bacterial species will have varying MICs where sensitive strains will have relatively low MICs, and resistant strains will have relatively high MICs (Turnidge and Paterson, 2007). Interestingly, ceftriaxone intrinsic resistant was found in strains Scott A and N1-227, while the reference strain ATCC 19116 showed fourfold adaptive resistance after sublethal chlorine exposure. Al-Nabulsi et al. (2015) demonstrated that antibiotic resistance in *L. monocytogenes* was increased after exposing it to 6% or 12% NaCl, reducing the pH to 5, and decreasing the temperature to 10°C. *L. monocytogenes* isolates from meat and dairy have also been found to be more resistant than its reference ATCC 19116 strain.

Stress adapted bacterial cells change their morphology and led to development of persisters, which may help in their ability to tolerate higher concentrations of antibiotics and antimicrobials (Levin and Rozen, 2006; Dawson et al., 2011). The development of persisters is observed under different conditions such as intracellular stress response (Dörr et al., 2009), oxidative stress response (Vega et al., 2012) and stringent response (Amato and Brynildsen, 2014). At sublethal concentrations, chlorine and RCS species can induce the SOS response, a state of temporary stop in bacterial cell division in bacteria and change to a long rod shape with multi-chromosomes containing long filaments. Bos et al. (2014) investigated the role of bud formation in antibiotic resistance propagation of *Escherichia coli* after sublethal ciprofloxacin exposure. In the present study, sublethal chlorine induced oxidative stress consistently induced persisters or multi chromosomal long rods in all three strains studied using TEM, which may aid in the increase of antibiotic tolerance. Our present findings of *L. monocytogenes* cell elongation and cell wall roughness as a bacterial response in stress adapted cells is in agreement with previous investigations (To et al., 2002). These results suggest a major role of outer member modulations in *L. monocytogenes* adaptive responses to antimicrobials and antibiotics (Ernst et al., 2001; Sallum and Chen, 2008). Similar to previous findings (Gray et al., 2014), we have also observed that *L. monocytogenes* cells also formed multi-chromosome filaments with buds in response to chlorine stress by electron microscopy studies.

## Conclusion

Our results show that the exposure to sublethal concentrations of chlorine could potentially have impact on the cross-responses of *L. monocytogenes* to commonly used antibiotics, enabling microorganisms to adapt to adverse environmental conditions and enhance antibiotic resistance. The changes in cell wall and membrane integrity resulting from the elongation of cells may contribute to possible routes of *L. monocytogenes* response for minor but significant increase in tolerance to chlorine and selective antibiotics.

## Author Contributions

The research was designed and led by RN from FSNHP and by CS and AK from POSC and was completed by MB while working for his Masters thesis research.

## Conflict of Interest Statement

The authors declare that the research was conducted in the absence of any commercial or financial relationships that could be construed as a potential conflict of interest.

## Supplementary Material

FIGURE S1 Transmission electron micrographs of *Listeria monocytogenes* N1-227 non-adapted **(A,B)** and oxidative stress adapted cells **(C–F)** gradually exposing to 375 ppm (3/4 MIC) from 250 ppm (1/2 MIC) of chlorine over 7 days. Micrographs represent planktonic cells: **(A,B)** control cells at different magnifications; **(C–F)** elongated cells and bud formation, indicated by arrow and multi-chromosome formation indicated by black circle in chlorine adapted cells at different magnifications.
