# Supplementary figures and images for: Listeria monocytogenes Response to Sublethal Chlorine Induced Oxidative Stress on Homologous and Heterologous Stress Adaptation
Source: Front Microbiol. 2018 Aug 31;9:2050. doi: 10.3389/fmicb.2018.02050 (PMC6127204; doi:10.3389/fmicb.2018.02050)

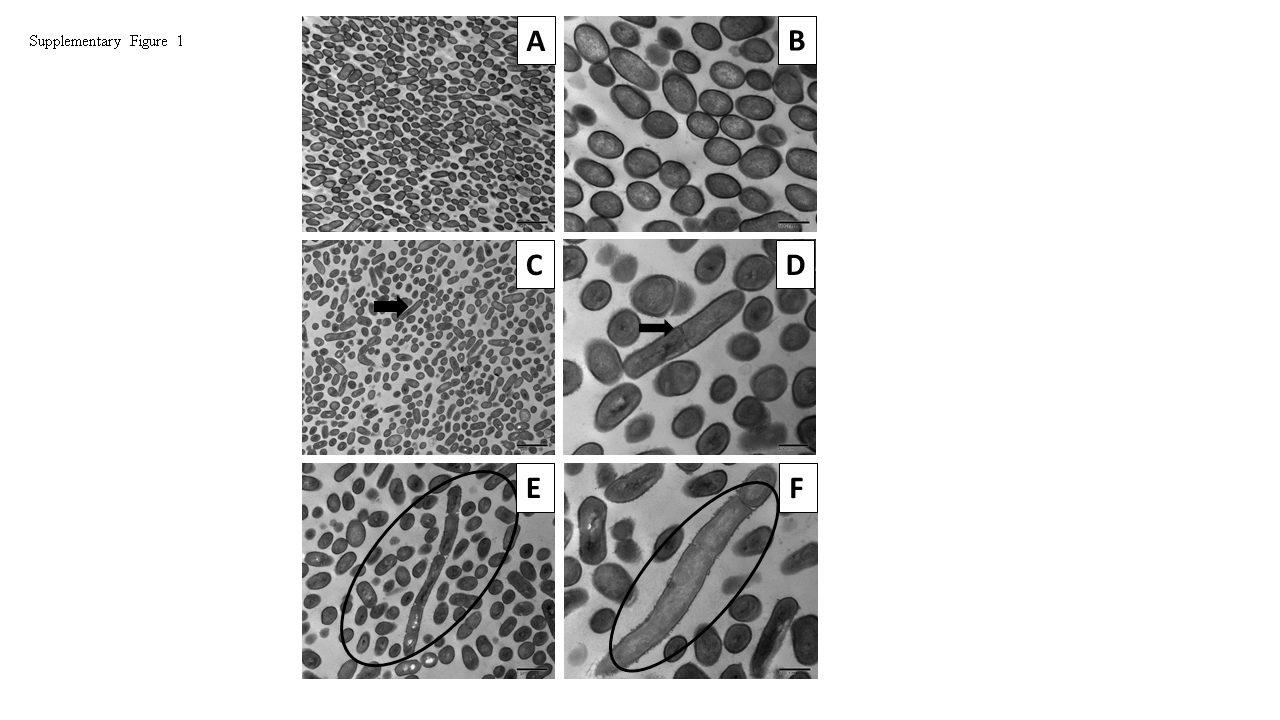

Supplement: FIGURE S1 — Transmission electron micrographs of Listeria monocytogenes N1-227 non-adapted (A,B) and oxidative stress adapted cells (C–F) gradually exposing to 375 ppm (3/4 MIC) from 250 ppm (1/2 MIC) of chlorine over 7 days. Micrographs represent planktonic cells: (A,B) control cells at different magnifications; (C–F) elongated cells and bud formation, indicated by arrow and multi-chromosome formation indicated by black circle in chlorine adapted cells at different magnifications. [file Image_1.TIF]
